# An Analysis of the Use of Anesthetic Blocks versus Local Anesthesia Infiltration in Primary Total Knee Arthroplasty Surgery

**DOI:** 10.3390/jcm13195706

**Published:** 2024-09-25

**Authors:** Silvia Gomez Gomez, Julián C. Segura Mata, José T. Alcalá Nalváiz, Felicito García-Álvarez García, Clara Marín Zaldívar, Amagoia Fernández de Gamarra Goiricelaya

**Affiliations:** 1 Department of Orthopedic and Trauma Surgery, Lower Limb Unit, MAZ Hospital, 50015 Zaragoza, Spain; 2 Department of Surgery, University of Zaragoza, 50009 Zaragoza, Spain; 3 Department of Statistical Methods, University of Zaragoza, 50009 Zaragoza, Spain; jtalcala@unizar.es; 4 Institute of Mathematics and Applications (IUMA), 50009 Zaragoza, Spain; 5 Instituto de Investigación Sanitaria Aragón (IIS Aragón), 50009 Zaragoza, Spain; 6 Department of Anesthesia, MAZ Hospital, 50015 Zaragoza, Spain

**Keywords:** enhanced recovery surgery in adults, postoperative pain, knee arthroplasty, analgesia, peripheral nerve block

## Abstract

**Objectives:** The aim of this study is to analyse the efficacy of using a combined infiltration between a popliteal artery and knee cap (IPACK) anaesthetic block and a selective saphenous nerve block compared to local infiltration with anaesthetic in knee replacement surgery. **Methods:** A retrospective observational study was conducted. A total of 312 patients who underwent primary total knee arthroplasty in our hospital between January 2019 and December 2022 were reviewed. Local intra-articular anaesthesia was used in 207 patients and combined nerve block in 105 patients (IPACK group). The mean age in the LIA group was 72.9 years and 70.4 years in the IPACK group. There were 44% men in the LIA group and 53.3% in the IPACK group. The primary outcome was the presence of poorly controlled pain requiring rescue opioid analgesia in the postoperative period. Secondary outcomes included pain scores, range of motion and length of hospital stay. **Results:** There were no significant differences in the age or gender distribution of patients between the two groups. One patient treated with anaesthetic blocks required rescue analgesia with opioids, while in the LIA group this occurred in 28.5% of cases. There were statistically significant higher VAS scores in the LIA group (*p* < 0.001). Range of motion was slightly greater in the block group (4.6°, *p* < 0.05). There were significant differences in hospital stay (2.4 days in the blocks group and 2.8 days in the LIA group (*p* < 0.05). **Conclusions:** In our series, patients treated with anaesthetic blocks showed better results with similar postoperative pain control. However, further studies are needed.

## 1. Introduction

Primary total knee arthroplasty (TKA) is one of the greatest therapeutic advances in the treatment of knee osteoarthritis. In addition, the demand for this type of surgery is increasing [1,2]. In recent years, surgical patients have benefitted greatly from advances in anaesthesia, pain control, minimally invasive surgery, and overall perioperative care [3,4,5].

TKA causes significant postoperative pain due to the surgical stress response, representing inflammatory, nociceptive and neuropathic pain, and may delay early mobilisation, complicate postoperative physiotherapy or even lead to other major complications. Therefore, adequate multimodal postoperative pain management is important [6]. In our hospital, all patients undergoing TKA received an information session approximately one month prior to surgery, with the aim of reducing the stress of the operation. In addition, a physiotherapist made a preoperative preparation to prepare the stabilising muscles or to improve the proprioception or the lack of it and the size of the perioperative damage. It is also used to reduce the use of opioids for postoperative pain control to reduce nausea and vomiting [7]. The multimodal approach to postoperative pain in TKA involves interventions at different stages of the perioperative process. These protocols vary widely between institutions, and there is currently no consensus [8]. 

There are many anaesthetic techniques described in the literature that have evolved over time [9]. Initially, local intra-articular anaesthesia (LIA) and femoral nerve block (FNB) were used, but FNB is associated with quadriceps weakness and risk of falling, and sciatic block with foot drop. To overcome these disadvantages, and thanks to the availability and knowledge of ultrasound techniques, more distal nerve block techniques have been developed, such as the saphenous nerve block in the adductor canal (ACB) and a combined infiltration between the popliteal artery and knee capsule (IPACK) [9]. Adductor canal block (ACB) is emerging as an alternative to femoral nerve block (FNB) because it provides analgesia to intra-articular and anteromedial parts of the knee while preserving quadriceps muscle strength. The IPACK block is emerging to avoid posterior knee pain, which is usually present after TKA due to the presence of posterior terminal sensory nerve branches.

The aim of this study is to analyse the efficacy of IPACK anaesthesia combined with selective saphenous nerve block versus LIA in knee replacement surgery. The rapid recovery protocol is the same for both groups; the only difference is the anaesthetic technique used in each group.

## 2. Material and Methods

A retrospective observational study was performed. This study has received approval from the Institutional Review Boards. It has been approved by the Ethics Committee of MAZ Hospital, by the Ethics Committee of Aragon (Spain) and is registered at ClinicalTrials.gov with ID NCT05338255. All patients who underwent a primary total knee replacement at our hospital between January 2019 and December 2022 were reviewed. Revision knee replacements and unicompartmental prostheses were excluded. Patients were divided into two groups according to whether they underwent local anaesthetic infiltration or anaesthetic block during surgery. The inclusion criteria were as follows: >18 of age, advanced knee osteoarthritis and patients who underwent primary total knee arthroplasty in our hospital between January 2019 and December 2022 and were included in American Society of Anesthesiologists (ASA) status I-III. The exclusion criteria were as follows: <18 years of age and ASA IV. There were 312 patients who met the inclusion criteria.

Between January 2019 and November 2021, 207 patients received LIA during surgery (LIA group), and between November 2021 and December 2022, 105 patients received the combined nerve block described above (anaesthetic nerve block group). In November 2021, the protocol of our hospital was updated and the performance of this type of anaesthetic block was introduced. From then on, all patients received this type of anaesthetic block instead of LIA. The mean age was 72.3 years (±8.3 standard deviation, SD). The mean age in the LIA group was 72.9 (±7.7 SD) years and in the block group 70.4 (±9.0 SD) years. There were 44% men in the LIA group and 53.3% in the IPACK group. The height (cm) and weight (kg) of all patients were recorded on enrolment. The degree of osteoarthritis was assessed according to the Alhback classification [10].

Prophylactic ischaemia was only used in cases of excessive bleeding during surgery: 8.8% of all cases. For knee prosthesis, Medacta’s GMK Sphere was used in 74.4% of patients. GMK Primary, also from Medacta, which is an ultra-congruent model of prosthesis, was used in 13.3% of patients. We found a variety of up to 6 different models and brands of primary prostheses in 12.3% of cases. Low molecular weight heparin (LMWH) prophylaxis was given for 45 days postoperatively. Antibiotic prophylaxis was given with cefazolin, with clindamycin in allergic patients. Follow-up was performed during the first postoperative month and complications were recorded.

All patients received a multimodal preoperative pain protocol, including oral paracetamol 1 g and celecoxib 400 mg the night before surgery. Patients were operated on under spinal anaesthesia. Skin disinfection was performed with povidone iodine. Lidocaine 1% (a local anaesthetic) was used for skin infiltration. Using a 25 or 27 G needle, 0.5% isobaric or hyperbaric bupivacaine was administered between the L3–L4 space without adrenaline, trying not to exceed the T8 level of sensation, and a maximum of 10 micrograms of intravenous fentanyl was used. In cases where spinal anaesthesia could not be performed due to technical difficulties or medical contraindications, general anaesthesia was used (7 patients), which was preferably total intravenous anaesthesia (TIVA). 

Between January 2019 and mid-November 2021, the local infiltration of anaesthesia (LIA) was performed during surgery according to the current protocol. This technique consisted of administering 150 mL in three phases during prosthesis implantation. In the first two phases, 0.2% ropivacaine (3.5 mg/kg dose, without exceeding toxic doses) and 1 mg adrenaline were administered at the level of the posterior knee capsule and arthrotomy. The final phase contained ropivacaine without adrenaline and was reserved for subcutaneous tissue.

In mid-November 2021, there was a change in the protocol, with the use of LIA being stopped in our hospital and ultrasound-guided anaesthetic blocks being adopted; these were IPACK blocks together with saphenous nerve blocks at the level of the adductor canal (ACB). The anaesthetic blocks were performed after spinal anaesthesia before surgery. First, an ultrasound-guided IPACK block was performed with 20 mL of ropivacaine 0.375%. Next, a selective ultrasound-guided saphenous nerve block was performed at the level of the adductor canal with 10 mL ropivacaine 0.375%.

All patients received prophylaxis for postoperative nausea and vomiting during surgery with 8 mg dexamethasone (4 mg in patients diagnosed with diabetes) and 4 mg ondansetron 30 min before the end of surgery. Perioperative analgesia consisted of 600 mg ibuprofen IV at the start of surgery and 1 g paracetamol IV at the end of surgery.

If the patient presented with pain in the post-anaesthesia recovery unit (URPA), despite the blocks or LIA, ibuprofen and paracetamol, the following rescue regimen was started in the immediate postoperative period: IV metamizole 2 g/12 h alternating with IV paracetamol 1 g/6 h. If there was more pain, tramadol 50 mg/12 h was given intravenously.

Analgesia in the postoperative period in the hospital ward consisted of IV paracetamol 1 g/8 h, IV dexketoprofen 50 mg/8 h and IV metamizole 2 g/8 h (the last one was only administered if there was some pain). On the second postoperative day, we switched from intravenous to oral analgesia: paracetamol 1 g/12 h, dexketoprofen 25 mg/8 h and metamizole 575 mg/8 h (the last one was only administered if there was some pain). If the patient had more pain, tramadol 50 mg/8 h was given intravenously. If the patient continued to have pain despite this rescue analgesia, the on-call physician could prescribe any rescue analgesia that was considered, including the use of morphic derivatives. All patients received a dose of 200 mg of celecoxib every 24 h after surgery.

Nurses assessed the level of postoperative pain in patients using the VAS at 24 and 48 h postoperatively. If the patient required analgesic rescue with tramadol or even a morphine derivative, this was all recorded on the patient evolution sheet for later evaluation.

The primary outcome was the presence of poorly controlled pain requiring rescue opioid analgesia in the postoperative period. Secondary outcomes included pain scores measured using a visual analogue scale (VAS), range of motion measured using a goniometer and length of hospital stay.

Statistical analyses were performed using R v.4.2.2, Spss v.29 and Jamovi v.2.3.18. Age, BMI and operative time were analysed using Student’s *t*-test. Gender and type of anaesthesia were analysed using a chi-squared test. Pain scores were compared using Student’s *t*-test. The Mann–Whitney U test was used to compare opioid requirements and other paired comparisons at each time interval. Continuous data are presented as mean (with standard deviation), and categorical data are presented as raw data and frequencies. The alpha level for our primary outcome was set at *p* < 0.05 and 95% confidence intervals (CI).

## 3. Results

Both groups are homogeneous in terms of demographic characteristics and therefore statistically comparable. There was no difference in gender distribution between the two groups (*p* = 0.117). Patient characteristics and surgical characteristics are shown in Table 1. The mean age in the LIA group was 72.9 (95% CI 71.9–74; 7.7 ± SD) years and in the blocks group 70.4 (95% CI 68.7–72.1; 9.0 ± SD) years. Body mass index (BMI) was 31.9 (95% CI 30.9–33.1; 4.4 ± SD) in the LIA group and 31.7 (95% CI 30.7–32.9; 4.6 ± SD) in the blocks group. 

There was also no significant difference between the type of anaesthesia technique (spinal anaesthesia was used in 97.9% of patients) and the degree of osteoarthritis according to Alhback’s classification (*p* = 0.501).

Looking at the operating times from skin to skin, the average time was 116 (23.1) min in the LIA group and 111 (24.1) min in the blocks group, which are statistically significant differences (*p* < 0.001). During surgery, 11 patients required rescue analgesia with morphine. All were in the LIA group, with statistically significant differences between the groups (*p* = 0.016). 

**Figure 1:**
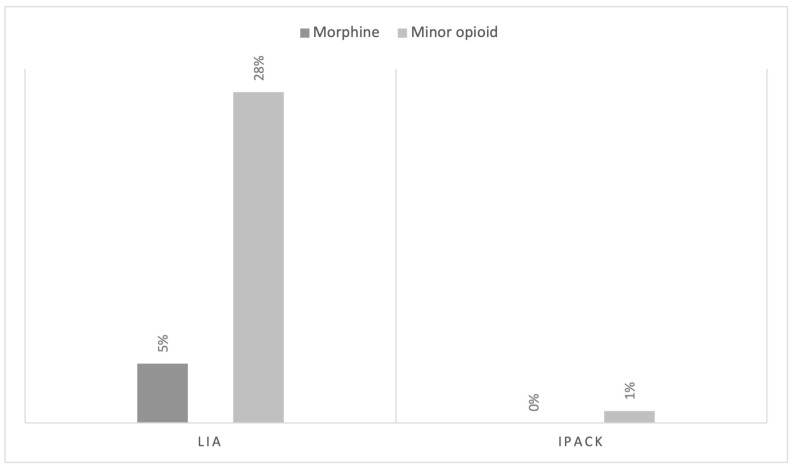
Rescue analgesia during surgery and in the hospital ward.

In hospital, only one patient treated with block anaesthesia required rescue analgesia with minor opioids after surgery (1%). A small percentage of patients treated with LIA required treatment with minor opioids such as tramadol (28.5%). There were statistically significant differences between the groups (*p* < 0.001). None required morphine or any other major opioid (Figure 1).

**Figure 2:**
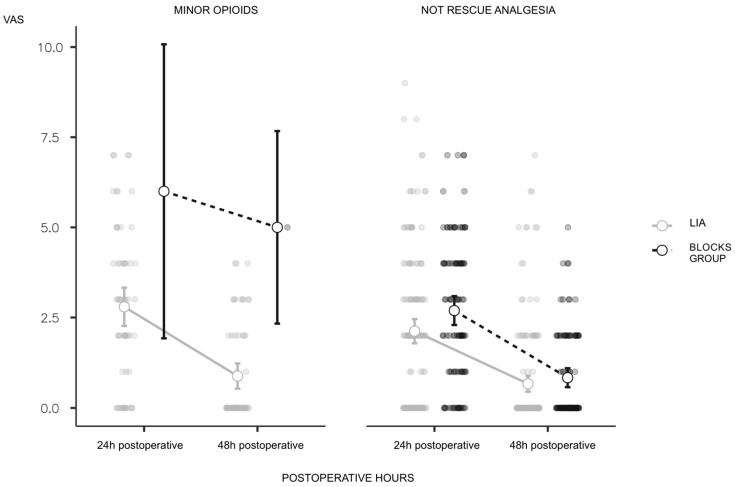
VAS evolution after surgery.

Overall, there were statistically significant differences between the first 24 h and first 48 h VAS (*p* < 0.001). The mean VAS was 2.5 on the first postoperative day and 0.8 on the second day. Although patients treated with anaesthetic blocks presented fewer data with elevated VAS, it is true that the mean values on the first postoperative day were higher compared to those treated with LIA (Figure 2). There were no statistically significant differences (*p* = 0.0964) between the VAS in the LIA and block groups in the first 24 h, although patients treated with LIA presented fewer elevated VAS data (mean 2.3 versus 2.7).

**Figure 3:**
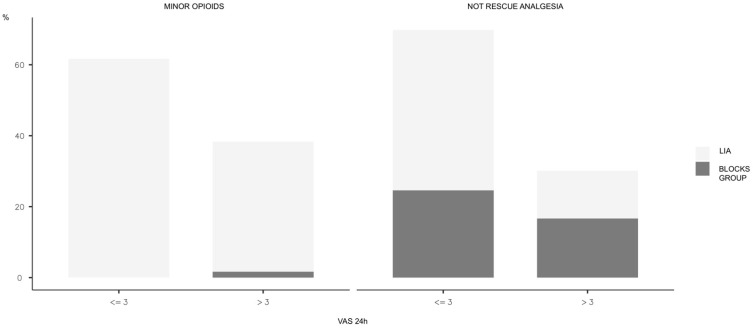
VAS 24 h.

Next, we will analyse the VAS data by creating two subgroups: VAS less than or equal to 3 (better pain control) and VAS greater than 3 (worse pain control). In this analysis, we do not consider the need for rescue analgesia. On the first postoperative day, 68.3% of patients had a VAS less than or equal to 3 (Figure 3). When analysing the different groups, 72.9% of patients treated with LIA had a VAS less than or equal to 3, compared to 59% in the block group (with statistically significant differences, *p* = 0.013). On the second postoperative day, there was no difference between the groups (*p* = 0.587); in fact, 94.2% of patients reported a VAS less than or equal to 3.

Range of motion was slightly greater, with a statistically significant difference of 4.6 degrees (*p* = 0.0017) for the block group. The mean range of motion for the LIA group was 83.1 (13) degrees and for the block group it was 87.7 (11.4) degrees. There were also statistically significant differences in hospital stay (2.4 days for the block group and 2.8 days for the LIA group, *p* = 0.0369). 

## 4. Discussion

In recent years, patients who have undergone TKA have benefitted from multimodal analgesia in the postoperative period. Multimodal analgesia provides superior pain relief, promotes knee recovery, and reduces opioid use and related adverse effects [11,12]. Protocols vary widely between centres and there is currently no consensus [13]. In other studies, 50% of patients undergoing TKA have moderate to severe pain [14] and approximately 20% of patients may be dissatisfied with their perioperative experience [15]. 

Memtsoudis SG et al. [16,17] recommended primary spinal anaesthesia for knee arthroplasty, along with peripheral nerve blocks. Anaesthetic blocks for pain control in knee arthroplasty have evolved over time. However, the femoral nerve block (FNB), long considered the gold standard, is associated with quadriceps weakness and risk of falling, and the sciatic block is associated with foot drop [9]. To overcome these disadvantages, more distal nerve block techniques have been developed, such as the saphenous nerve block in the adductor canal [18]. Perhaps the reason for the establishment of this technique is the availability and knowledge of ultrasound techniques.

More recently, the adductor canal block, together with the IPACK block, has gained popularity with the expected benefit of providing analgesia to both the anterior and posterior knee [19]. However, it is not yet clear whether the adductor canal block combined with the IPACK block is more effective than LIA in controlling pain after total knee arthroplasty [20]. We aimed to analyse the efficacy of using a combined infiltration between the popliteal artery and knee capsule (IPACK) anaesthetic block together with a selective saphenous nerve block versus local anaesthetic infiltration in knee replacement surgery.

The demographic characteristics of our patients were homogeneous, representative of the population sample and therefore comparable with those of other studies. For example, Ochroch J et al.’s [21] study had a mean age of 67.7 years (±7.8) (slightly lower than ours) and a BMI of 31.9 (the same as ours), while 75% of the patients underwent spinal anaesthesia (lower than ours) and the operation time was 76 min (lower than ours). Holzapfel DE et al. [22] described an average age of 66.1 years (±10.7), a BMI of 29 (±5.5), an operation time, in minutes, of 76.2 (±30.9) and a length of stay of 8.9 (±3.0) days (higher than the average). Trueba-Davalillo C et al. [23], who carried out a comparative study using ischaemia until implantation, ischaemia throughout the operation or no ischaemia, reported operating times of 120, 130 and 140 min, respectively. The large difference between the surgical times reported in the literature and ours is striking. In many cases, it is not specified what time was measured and sometimes it is not specified whether the operation was performed with or without ischaemia. It is clearly reported in the literature that performing surgery with ischaemia reduces operative time [24], which could explain these results. However, the reduction in operating time is the only advantage of using ischaemia today (against many disadvantages such as an increased risk of adverse events, severe pain, decreased postoperative joint balance and a prolonged hospital stay). Therefore, its routine use for this type of procedure is not justified [25].

Hussain N et al. [26], in their systematic review and meta-analysis, conclude that adding an IPACK block to ACB in the setting of periarticular LIA does not improve analgesic outcomes after TKA. In the absence of LIA, the addition of IPACK to ACB reduces pain for up to 24 h and improves functional recovery. Their results do not support the addition of these blocks when LIA is routinely administered. Two years after this study, they published another systematic review suggesting that continuous ACB, but not single-injection ACB and/or single-injection IPACK, may provide statistically superior analgesia when added to LIA for TKA compared with LIA alone [27]. Wang CG et al. [28] presented a randomised, double-blind, controlled trial. They conducted a study comparing two groups: ACB + IPACK versus ACB. They conclude that the combination of ACB + IPACK blocks could reduce the incidence of moderate to severe posterior knee pain, improve postoperative pain in the first 24 h after knee arthroplasty and promote the recovery of motor function. However, the addition of IPACK to ACB did not reduce opioid consumption. Tang X et al. [29] concluded that the addition of IPACK to ACB improved pain scores, morphine consumption and functional ability. In our case, since the comparison was LIA with IPACK + ACB, a decrease in the consumption of major and minor opioids was observed.

Qiao Y et al. [30] analysed IPACK block supplementation in addition to standard postoperative analgesia and concluded that it can be used effectively and safely to relieve early postoperative pain after total knee arthroplasty. IPACK supplementation significantly reduced rest pain scores after TKA, reduced postoperative opioid consumption within 24 h and total opioid consumption, and could reduce the length of stay. These results are consistent with ours. Kampitak W [31] concluded that the combination of IPACK with continuous ACB in multimodal analgesia relieved pain with movement better than the IPACK block alone during the 8 h postoperatively after TKA. Roy R [32] suggested the use of the 4-in-1 modified block, a new technique that is comparable to the combined ACB + IPACK technique in terms of analgesic control. In any case, more studies are needed to better analyse the results of the different types of anaesthetic blocks, as we found a great variety in the techniques described in the bibliography. 

Laoruengthana A et al. [33] analysed the differences between the use of IPACK + ACB blocks and LIA. Their findings concluded that the VAS and morphine requirements after TKA of the IPACK + ACB group were comparable to the LIA group during the first 48 h and that the knee flexion angle was not significantly different between the groups. We found differences in the postoperative range of motion in our study. This may be due to the different amounts of analgesia administered (LIA or blocks), the fact that the surgery was performed without ischaemia [22] or perhaps that the prosthesis model played a role. GMK Sphere is an innovative total knee implant designed to provide maximum functional stability, with the aim of increasing patient satisfaction with total knee arthroplasty in activities of daily living and reducing postoperative knee pain [34,35]. It may be a combination of all of these factors, but more research is needed.

In terms of hospitalisation, the literature reports a reduction from 6 to 8 days to an average of 2 days thanks to multimodal rehabilitation programmes [36], according to our results. The possibility of performing these interventions on an outpatient basis is now being considered [37]. Further studies are needed to analyse this question.

## 5. Conclusions

In our series, patients treated with anaesthetic blocks showed better results with similar control of postoperative pain. However, further studies are needed. This article is about a new technique that is useful and opens a new scope of studies on related topics. This article will also contribute to reach a consensus in establishing a pain management protocol in TKA patients, especially when there is a lack of consensus on multimodal analgesia protocol between institutions.

## Figures and Tables

**Table 1:** Patients’ characteristics and surgical characteristics.

Category		LIA (*n* = 207)	ACB + IPACK (*n* = 105)	*p* Value
Age (years)		72.9 (±7.7)	70.4 (±9.0)	
Gender	Men	44%	53.3%	0.117
BMI		31.9 (±4.4)	31.7 (±4.6)	
Ahlback classification	II	7%	10.9%	0.501
	III	47.8%	47.5%	
	IV	45.2%	41.6%	
Surgery time		116 (±23.1)	111 (±24.1)	<0.001
Model of prosthesis: GMK Sphere		64.7%	93.3%	<0.001
Rescue with morphine (during surgery)		5.3%	0%	0.016
Rescue with minor opioids (hospital ward)		1%	28.5%	<0.001
VAS 24 h		2.3 (±2.1)	2.7 (±2.0)	0.0964
VAS 48 h		0.7 (±1.4)	0.8 (±1.3)	0.6323
Range of motion (degrees)		83.1 (±13)	87.7 (±11.4)	0.0017
Length of stay (days)		2.8	2.4	0.0369

LIA, local intra-articular anaesthesia; ACB, adductor canal block; IPACK, interspace between the popliteal artery and the capsule of the posterior knee; BMI, body mass index; VAS, visual analogue scale.

## Data Availability

The datasets used belong to the MAZ hospital.
